# The cultural safety of research reports on primary healthcare use by Indigenous Peoples: a systematic review

**DOI:** 10.1186/s12913-024-11314-3

**Published:** 2024-07-31

**Authors:** Amandi Hiyare-Hewage, Victoria Sinka, Eleonora Dal Grande, Marianne Kerr, Siah Kim, Kylie-Ann Mallitt, Michelle Dickson, Allison Jaure, Rhonda Wilson, Jonathan C. Craig, Jacqueline H. Stephens

**Affiliations:** 1 Flinders Health and Medical Research Institute, College of Medicine and Public Health, Flinders University, Adelaide, Australia; 2 Sydney School of Public Health, Faculty of Medicine and Health, The University of Sydney, Sydney, Australia; 3 The Poche Centre for Indigenous Health, Faculty of Medicine and Health, The University of Sydney, Sydney, Australia; 4 Centre for Kidney Research, The Children’s Hospital at Westmead, Sydney, Australia; 5 Department of Nursing, RMIT University, Melbourne, Australia

**Keywords:** Indigenous health, Primary healthcare, Chronic disease, Cultural safety, Dissemination

## Abstract

**Introduction:**

Community-driven research in primary healthcare (PHC) may reduce the chronic disease burden in Indigenous peoples. This systematic review assessed the cultural safety of reports of research on PHC use by Indigenous peoples from four countries with similar colonial histories.

**Methods:**

Medline, CINAHL and Embase were all systematically searched from 1st January 2002 to 4th April 2023. Papers were included if they were original studies, published in English and included data (quantitative, qualitative and/or mixed methods) on primary healthcare use for chronic disease (chronic kidney disease, cardiovascular disease and/or diabetes mellitus) by Indigenous Peoples from Western colonial countries. Study screening and data extraction were undertaken independently by two authors, at least one of whom was Indigenous. The baseline characteristics of the papers were analyzed using descriptive statistics. Aspects of cultural safety of the research papers were assessed using two quality appraisal tools: the CONSIDER tool and the CREATE tool (subset analysis). This systematic review was conducted in accordance with the Assessing the Methodological Quality of Systematic Reviews (AMSTAR) tool.

**Results:**

We identified 35 papers from Australia, New Zealand, Canada, and the United States. Most papers were quantitative (*n* = 21) and included data on 42,438 people. Cultural safety across the included papers varied significantly with gaps in adequate reporting of research partnerships, provision of clear collective consent from participants and Indigenous research governance throughout the research process, particularly in dissemination. The majority of the papers (94%, 33/35) stated that research aims emerged from communities or empirical evidence. We also found that 71.4% (25/35) of papers reported of using strengths-based approaches by considering the impacts of colonization on reduced primary healthcare access.

**Conclusion:**

Research on Indigenous PHC use should adopt more culturally safe ways of providing care and producing research outputs which are relevant to community needs by privileging Indigenous voices throughout the research process including dissemination. Indigenous stakeholders should participate more formally and explicitly throughout the process to guide research practices, inclusive of Indigenous values and community needs.

**Supplementary Information:**

The online version contains supplementary material available at 10.1186/s12913-024-11314-3.

## Background

Prior to colonization, Indigenous Peoples across Australia, New Zealand, Canada, and the United States lived self-determined lives for tens of thousands of years. [1, 2] As a result of ongoing colonizing practices, Indigenous Peoples continue to experience systemic racism, geographical remoteness, intergenerational poverty, exclusion from Western models of health and limited access to primary healthcare (PHC) services all of which exacerbate health inequity leading to a higher prevalence of chronic disease. [2] Without early detection and preventive healthcare, chronic disease can lead to severe comorbidities and in some cases premature mortality. [3, 4] In Australia, the gap in life expectancy between Aboriginal and Torres Strait Islander Peoples and non-Indigenous Peoples is 12 years for males and 10 years for females, with chronic diseases, such as chronic kidney disease, cardiovascular disease, and diabetes mellitus, known as major contributors to this gap. [5] In the years 2016-20, the two main underlying causes of death for Aboriginal and Torres Strait Islander Peoples were also coronary heart disease and diabetes mellitus. [6] This gap can be eliminated by privileging Indigenous voices in PHC services and research outputs to ensure PHC meets community needs. [7] Culturally unsafe practices that disempower and exploit Indigenous Peoples’ identity within previous PHC practices have been a barrier to preventive PHC. [8, 9] Indigenous health research is an important tool to identify, monitor, and address enablers of PHC access. As such, culturally safe practices in research processes, including the reporting of research, must be prioritised. [10]

Introduced in the early 1990s by a group of Māori nurses, the concept of ‘cultural safety’ is to ensure Indigenous cultural values, strengths and differences are respected and the impacts of colonization, such as racism and inequity, are addressed. [11] Furthermore, the integration of cultural safety in healthcare practices in an active manner reconfigures health services to provide greater equity of realised access. [12] There has also been an increase in recognition in the involvement of Indigenous Peoples in research processes internationally to produce outputs that are culturally safe and collaborative [13, 14]. Indigenous data sovereignty is a global movement concerned with the rights of Indigenous Peoples to exercise ownership over Indigenous data. [15] Data is a cultural and economic asset for Indigenous Peoples and changing the narrative of PHC research by enhancing Indigenous data sovereignty and utilizing Indigenous research governance results in research with higher relevance and benefit to communities. [14] Therefore, this systematic review aimed to assess the cultural safety of reports of research on primary health care service use by Indigenous Peoples with chronic disease (chronic kidney disease, cardiovascular disease and/or diabetes mellitus) from Western countries sharing similar colonial histories, specifically Australia, Canada, New Zealand, and the United States. [1]

## Methods

This systematic review was structured according to the Preferred Reporting Items for Systematic Reviews and Meta-Analyses statement guidelines. [16] The protocol was registered with PROSPERO [Registration number CRD42022318565]. We report this systematic review following the ‘Assessing the Methodological quality of Systematic Reviews’ (AMSTAR) guidelines. [17]

### Eligibility criteria

To be deemed eligible, research papers (either quantitative, qualitative and/or mixed methods) had to report on PHC service use (general practice, nurse, and Indigenous health services) by Indigenous populations within Australia (Aboriginal, Torres Strait Islander), United States (Native American), Canada (First Nations, Inuit, Métis) or New Zealand (Māori). Full-text papers were included if published in English and since January 2002; this publication date was chosen as this corresponds with when the concept of ‘cultural safety’ became more widely used. [11]

### Search strategy

The following databases were searched from 1 January 2002 to 4 April 2023 for keywords and MeSH headings: OVID Medline, CINAHL and OVID Embase. An initial search was conducted in Medline to identify search results and assist in refining key terms. The final search terms incorporate concepts of *chronic disease* (*chronic kidney disease*,* cardiovascular disease*,* diabetes mellitus)*,* primary health care and Indigeneity.* Full search strategies are included in the Supplemental Material.

### Selection of sources

**Fig. 1:**
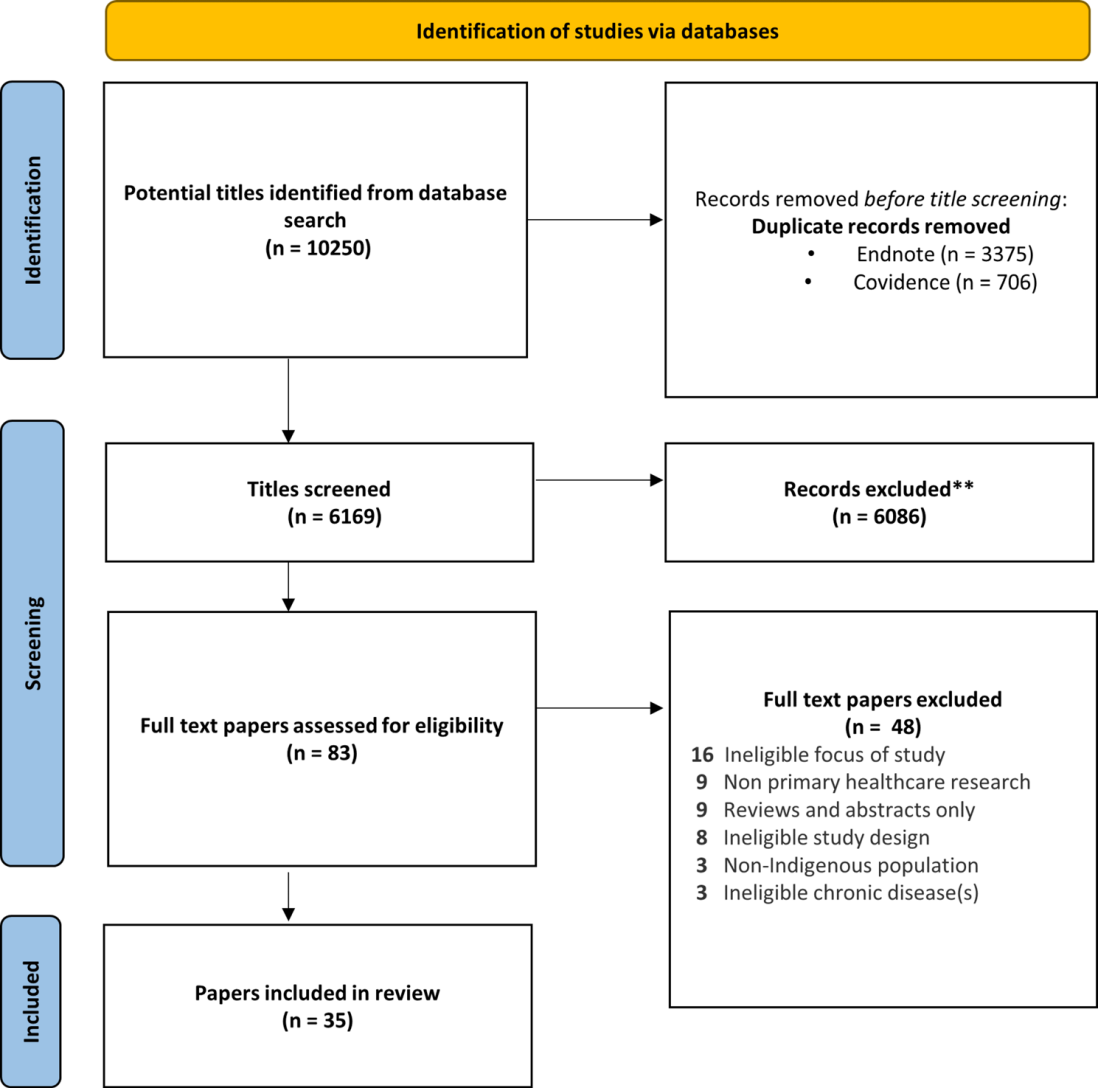
PRISMA flow chart

Initially, the search results were imported into Endnote to remove duplicates and then into Covidence, a screening and data extraction tool to remove any further duplicates (Fig. 1) [18]. Title and abstracts were screened in duplicate independently by three systematic reviewers (AH, VS, MK). All full-text screening (*n* = 82) was performed by one reviewer (AH) and a second reviewer (SK) conducted a full-text review of 29% of the included papers (*n* = 10/35) with 100% agreement (in accordance with AMSTAR guidelines). [17] Full texts were assessed in detail according to the inclusion criteria (Table 1) with exclusion reasons documented.

**Table 1:** Inclusion and exclusion criteria

Category	Included	Excluded
Study Type	Either quantitative, qualitative, or mixed methods research.	Editorials, reviews and abstracts.
Outcome of Interest	Primary healthcare (general practice, nurse, Indigenous health services) use for populations of interest with chronic disease (chronic kidney disease, diabetes mellitus, cardiovascular disease)	• Other levels of health care (e.g. secondary) • Other health conditions (e.g. cancer, skin conditions, ear health)
Populations of Interest	Indigenous populations within Australia (Aboriginal, Torres Strait Islander), United States (Native American), Canada (First Nations, Inuit, Métis) or New Zealand (Māori).	Research not including Indigenous populations within Australia, United States, Canada or New Zealand
Publication Dates	Published after January 2002	Published before January 2002
Publication Language	Only English	Languages other than English

### Data extraction

Data extraction was performed using Covidence software by two reviewers (AH, EDG) with conflicts resolved through consensus by the senior author (JS). A data extraction template was developed, informed by a previous review, and revised, updated, and piloted before being finalised for use. [19] Data extracted included lead author name, year and country of publication, chronic disease of interest, number of participants, and number of Indigenous participants (Table 2). Data extraction was exported from Covidence for analysis.

**Table 2:** Characteristics of included papers (*n* = 35)

Study details	Country	Chronic disease(s) of interest	Type of study	Sample size (*N*)	Number of Indigenous participants (*n*, %)
Rhoades et al., 2003 [25]	United States	Cardiovascular Disease; Chronic Kidney Disease; Diabetes Mellitus	Quantitative study	524	524 (100%)
Maple-Brown et al., 2004 [31]	Australia	Diabetes Mellitus	Quantitative study	595	595 (100%)
Sinclair et al., 2006 [64]	New Zealand	Cardiovascular Disease; Diabetes Mellitus	Quantitative study	3516	N/S
Si et al., 2006 [32]	Australia	Diabetes Mellitus	Mixed-methods study	137	137 (100%)
Thomas et al., 2007 [46]	Australia	Diabetes Mellitus	Quantitative study	593	144 (24.3%)
Hotu et al., 2010 [45]	New Zealand	Chronic Kidney Disease; Diabetes Mellitus	Quantitative study	65	N/S
Lawrenson et al., 2010 [48]	New Zealand	Diabetes Mellitus	Quantitative study	300	249 (83%)
Spurling et al., 2010 [33]	Australia	Diabetes Mellitus	Mixed-methods study	132	N/S
Mehta et al., 2011 [30]	New Zealand	Cardiovascular Disease	Quantitative study	7285	1556 (21.4%)
Burgess et al., 2011 [34]	Australia	Cardiovascular Disease	Quantitative study	64	64 (100%)
Faatoese et al., 2011 [24]	New Zealand	Cardiovascular Disease	Quantitative study	252	252 (100%)
Aspin et al., 2012 [22]	Australia	Cardiovascular Disease; Chronic Kidney Disease; Diabetes Mellitus	Qualitative study	19	19 (100%)
Shaw et al., 2013 [43]	United States	Diabetes Mellitus	Qualitative study	13	13 (100%)
Artuso et al., 2013 [35]	Australia	Cardiovascular Disease	Qualitative study	34	7 (21%)
Cuesta-Briand et al., 2014 [65]	Australia	Diabetes Mellitus	Qualitative study	38	18 (47.4%)
Chung et al., 2014 [36]	Australia	Diabetes Mellitus	Quantitative study	65	55 (84.6%)
Sheridan et al., 2015 [66]	New Zealand	Cardiovascular Disease; Diabetes Mellitus; COPD, depression, arthritis, gout	Qualitative study	42	8 (19%)
Smith et al., 2015 [4]	United States	Diabetes Mellitus	Quantitative study	2138	N/S
Liu et al., 2015 [37]	Australia	Cardiovascular Disease	Qualitative study	94	19 (20.2%)
Schierhout et al., 2016 [38]	Australia	Diabetes Mellitus	Quantitative study	15,622	N/S
Askew et al., 2016 [39]	Australia	Cardiovascular Disease; Chronic Kidney Disease; Diabetes Mellitus	Mixed-methods study	37	37 (100%)
Jacklin et al., 2017 [51]	Canada	Diabetes Mellitus	Qualitative study	32	32 (100%)
King et al., 2018 [26]	United States	Diabetes Mellitus	Quantitative study	2661	N/S
Hu et al., 2019 [40]	Australia	Cardiovascular Disease; Chronic Kidney Disease; Diabetes Mellitus	Quantitative study	815	294 (36.1%)
Barton et al., 2019 [29]	Australia	Diabetes Mellitus	Mixed-methods study	21	N/S
Franz et al., 2020 [50]	United States	Diabetes Mellitus	Quantitative study	3053	173 (0.06%)
Askew et al., 2020 [41]	Australia	Cardiovascular Disease; Chronic Kidney Disease; Diabetes Mellitus	Mixed-methods study	60	60 (100%)
Wood et al., 2020 [47]	Australia	Diabetes Mellitus; Hyperglycaemia post-pregnancy	Quantitative study	197	188 (95.4%)
Tane et al., 2021 [44]	New Zealand	Diabetes Mellitus	Qualitative study	32	13 (40.6%)
Brazionis et al., 2021 [42]	Australia	Diabetes Mellitus	Quantitative study	301	301 (100%)
Moore et al., 2022 [27]	United States	Diabetes Mellitus	Quantitative study	2635	1564 (59.4%)
Eer et al., 2022 [23]	Australia	Diabetes Mellitus	Quantitative study	126	113 (89.7%)
Atkinson-Briggs et al., 2022 [49]	Australia	Diabetes Mellitus	Quantitative Study	135	NS
Schaefer et al., 2022 [28]	United States	Cardiovascular Disease	Qualitative study	16	16 (100%)
Lakhan et al., 2022 [67]	Australia	Chronic Kidney Disease	Quantitative Study	1181	1181 (100%)

NS: Not specified; N/A: Not applicable

### Cultural safety assessment

The cultural safety of the reporting within included papers was assessed using a validated assessment tool - the Consolidated Criteria for Strengthening Reporting of Health Research Involving Indigenous Peoples (the CONSIDER tool). [20] The CONSIDER tool consists of eight research domains incorporating 17 criteria for reporting research involving Indigenous Peoples. The eight domains are (1) governance; (2) relationships; (3) prioritization; (4) methodologies; (5) participation; (6) capacity; (7) analysis and findings; and (8) dissemination, and they address aspects of cultural safety. This tool was developed after a collaborative prioritisation process of reviewing research guidelines about Indigenous health research from seven nations of which four are included in this review (Canada, United States, New Zealand, and Australia). One reviewer (AH) conducted cultural safety assessment for all papers (*n* = 35) using the CONSIDER tool, with a subset (25%) assessed by an Indigenous author (VS) to ensure accuracy. Where data was missing or unclear the researchers contacted the corresponding author to retrieve additional information. A subset assessment of papers reporting research from Australia (*n* = 20/35) was performed using the Aboriginal and Torres Strait Islander Quality Appraisal (CREATE) Tool. [21] The CREATE tool was developed specifically for Australian papers and therefore, not appropriate to assess papers from other countries.

### Data analysis

Descriptive analyses including means, frequencies and proportions were performed using R Studio (2020. RStudio, PBC, Boston, MA URL http://www.rstudio.com/). The reporting of aspects of cultural safety for the included papers were categorically synthesised using the different domains in the CONSIDER statement and CREATE tool.

### Research governance

This review was conducted as part of the Antecedents of Renal Disease in Aboriginal Children (ARDAC) research program. The review was conducted with input from the ARDAC Advisory group, which comprises Aboriginal and Torres Strait Islander stakeholders and investigators. Input from the Advisory Group was sought throughout the research process, including the design of the research question, selecting relevant cultural safety assessment tools, and development of the search strategy to ensure Indigenous stakeholder input. Findings from this systematic review were presented at both the Advisory Group and Investigator meetings with feedback incorporated accordingly. Dissemination and implementation of the research findings will be undertaken with further input from the Advisory Group and other Aboriginal and Torres Strait Islander Community members to ensure the findings are translated into healthcare policy in culturally appropriate ways.

## Results

### Sources of evidence

On 4 April 2023, a total of 10,250 papers were identified during the database search (Fig. 1). After the removal of duplicates, 6,169 papers were screened. Following title and abstract screening, 82 papers were identified for full-text review, with 35 papers meeting the inclusion criteria and included in this systematic review.

### Characteristics of included papers

Of the 35 included papers, the majority reported research conducted in Australia (57%, 20/35), followed by New Zealand (20%, 7/35), United States (20%, 7/35) and Canada (2.9%, 1/35). Most papers used quantitative methods (60%, 21/35), followed by qualitative (29%, 10/35) and mixed methods (11%, 4/35). In total, the papers presented data on 42,438 peoples (median: 132, range: 13–15,000). The majority reported on PHC use by Indigenous peoples with diabetes mellitus (57%, 20/35), with only one paper (2.9%, 1/35) reporting on PHC use by Indigenous peoples with chronic kidney disease.

### Cultural safety assessment

**Fig. 2:**
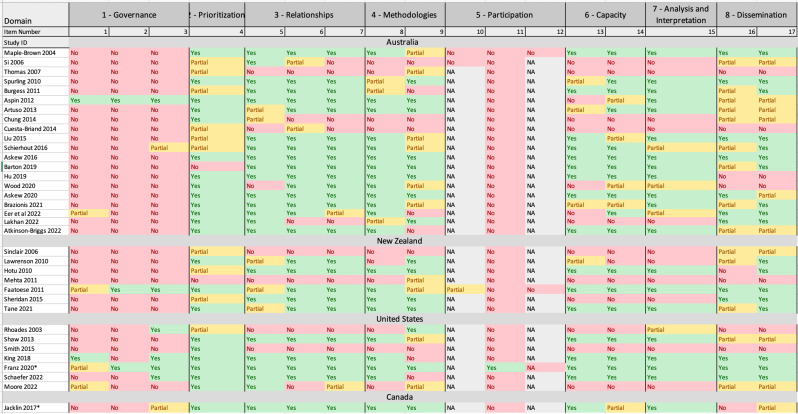
Results of the cultural safety assessment of included studies using the CONSIDER tool. *Footnotes* Please note papers were shaded on the reporting of aspects of cultural safety using the CONSIDER tool. Where, green = yes, red = no, yellow = partial and grey = not applicable (NA)

The results from cultural safety assessment using the CONSIDER Statement are presented in Fig. 2 for the included papers. These results varied across the four countries for each of the eight research domains in CONSIDER Statement and are discussed in detail below. The CREATE assessment methods and results are presented in the Supplementary Material.

### Domain 1 – research governance

Overwhelmingly, reporting of PHC research required further detail on *research governance* with just 17% (6/35) papers included adequate reporting of the research relationship. [22, 23] For example, the informal agreements through MOU (Memorandum of Understanding) or MOA (Memorandum of Agreement), that occurred between research institutions hosting the research and the Indigenous organisations with oversight responsibilities to the participants and communities involved in the research. Only 8.6% (3/35) provided a statement addressing harm minimisation and protection of Indigenous intellectual property and knowledge arising from the research [22, 24]. One paper clearly detailed this by stating the aims of partnership between researchers and community to avoid errors of non-partnered research with Indigenous Peoples. [22] Around 22.9% (8/35) papers addressed the protection of Indigenous intellectual property and knowledge. [22, 24–28]

### Domain 2 - prioritisation

For *prioritisation*, 94%, (33/35) of papers reported that the research aims emerged from either community driven priorities and/or empirical evidence with only two papers not reporting this in the research outputs [29, 30]. These 33 papers clearly identified and outlined whether Indigenous stakeholders, including individuals and communities, participated in the identification of research aims or whether existing evidence such as health data or priorities determined by health policies led to development of research aims.

### Domain 3 – relationships

The *relationships* domain refers to partnerships between Indigenous stakeholders, participants, and the research team. Overall, for this domain, most of the papers across the four countries performed well. Many of the papers (80%, 28/35) reported honouring Indigenous ethical guidelines and obtaining ethical approvals from relevant Indigenous ethics committees with only 20% (8/35) papers lacking the detail of this. [22, 23, 29, 31–42] However, these eight papers did include ethical approval but from non-Indigenous organisations.

### Domain 4 – methodologies

For *methodologies*, 71.4% (25/35) of the papers mentioned some description of the methodological approach which include Indigenous quantitative and qualitative methods that have known positive impacts on Indigenous stakeholders. For example, one study clearly outlined this by stating that questions aligning with tribally determined health priorities and corporate objectives established by the Indigenous leaders were asked in the study. [43] Other studies also provided detail about utilising culturally appropriate models of health relevant to study objectives [44, 45]. However, clearer detail of using specific Indigenous research methodologies needs to be included and/or considered to ensure research conducted on Indigenous Peoples is moving away from biased Western research methodologies. Further, 71.4% (25/35) of the papers reported some consideration of the physical, social, economic, and cultural environment [22, 23, 29, 31, 33, 35, 37–42, 46–48]. For example, these papers mentioned the impacts of ongoing colonizing practices such as racism and resulting social disadvantage as being risk factors for chronic disease outcomes for Indigenous Peoples.

### Domain 5 – research participation

*Research participation* covers ethical considerations of the data gathered including data confidentiality, the burden of research participation on Indigenous communities, and future use of Indigenous data and knowledge. This domain also includes consideration of consent, storage, and access of biological samples. To ensure data privileges Indigenous knowledges and meets current and future needs, the data collected on Indigenous Peoples need to belong to the community and relevant Indigenous stakeholders which they are derived from. Most of the papers (91%, 32/35) included in this review did not obtain blood samples and therefore, the items from this domain were not applicable. Three papers did mention the use of blood samples in the study, however, the storage of these samples and the process of removal from traditional lands were not specified in the papers. [24, 31, 32] Researchers should ensure that any samples taken away from traditional lands (if done) needs to be discussed frankly as part of the research agreement. Further, whilst most papers in this review were using quantitative research methods, most of the data were de-identified and from hospital records or survey data and therefore, item 12 was not applicable to most studies given that the data had been collected prior to the study and not as part of the study.

### Domain 6 – capacity

For *capacity*, 60% (21/35) of papers provided some explanation of Indigenous research capacity such as working with Indigenous stakeholders and providing training opportunities with only 49% (17/35) of the papers fully detailing this in the research outputs. [22–24, 26, 28, 29, 31, 33–35, 37–45, 47, 49–51] For example, these 17 papers mentioned either employment of Indigenous staff to undertake analysis in culturally appropriate ways and to maintain relationships between communities appropriately. These 17 papers also outlined whether training opportunities provided to Indigenous researchers as part of the project to strengthen research capacity were undertaken. However, further detail is required to clearly state Indigenous researcher’s position within the study, outline whether they hold seniority positions within the study to enhance self-determination. Around 66% (23/35) articles reported professional development by the research team to develop a capacity to partner with Indigenous peoples. Examples within included papers are reporting of any culturally safe training undertaken by researchers and some statements which recognises Indigenous values within the research.

### Domain 7 – analysis and interpretation

For *analysis and interpretation*, 68.6% (24/35) of the papers provided some detail about how the research analysis and reporting support critical inquiry and a strengths-based approach which was inclusive of Indigenous value. For example, one of these studies mentioned that research analysis method fostered daily reflection and honoured Indigenous ways of knowing and sharing. [51] Whilst another study mentioned that models of health employed by the study were informed by Aboriginal and/or Torres Strait Islander conceptualisations of health given that these models have the potential to improve biomedical and psychosocial health status. [39]

### Domain 8 – dissemination

Lastly, it is widely understood that *dissemination* of research is essential to achieve social, economic, and political impact. The papers included varied in detailing how research teams disseminate their research outcomes to appropriate Indigenous stakeholders which were parallel with standard pathways. Only 34% (12/35) of papers provided a detailed description of the dissemination of research findings to relevant Indigenous governing bodies and peoples [31]. However, 80% (28/35) of papers provided some process of knowledge translation and implementation to support Indigenous advancement. One study mentioned the development of patient coaching materials which patients can use within their homes. [50] This study also emphasised the importance of meeting with advisory groups and communities to ensure study findings are disseminated in a comprehensible manner to patients and families.

## Discussion

This systematic review has found reporting of research on Indigenous PHC use has not always been done in a culturally safe manner and that Indigenous voices need to be consistently and adequately included in PHC research. Of note, the reporting of aspects of cultural safety of the 35 included papers in this review varied significantly. Our findings reveal that research governance and data sovereignty in PHC research involving Indigenous Peoples has not always been reported adequately by researchers.

Indigenous research governance minimises the potential harm to Indigenous Peoples by fostering relationships that maximises the benefits of research in Indigenous primary health service use. Ensuring that partnership agreements between research institutions and the Indigenous organisation are clearly outlined in the research paper enhances cultural safety and recognises the centrality of Indigenous leadership in research conduct. In interviewing 60 participants, an Australian qualitative study aimed to identify whether community engagement in healthcare was effective. [52] Findings from their study suggested that community owned and driven healthcare decisions improved healthcare and led to increased healthcare access, thus highlighting the importance of Indigenous research governance within health services research. [52] Findings from Burchill et al. mentioned Indigenous research governance requires fundamental re-orientation and investment to give control of the framing, design and conduct of Indigenous health research to Indigenous Peoples. [53] Only 17% of the included papers reported on research governance or details of partnership agreements with Indigenous communities. Primary healthcare services are considered the frontline for health care delivery and providing detail of Indigenous leadership within this area of research enhances acceptability of research findings within communities and contributes to improved PHC service use.

The impact of colonization has resulted in Indigenous Peoples being isolated from the language, control, and production of data relating to them. [54] The United Nations Declaration on the Rights of Indigenous Peoples in 2007 outlined the importance of data sovereignty as a way for Indigenous Peoples to remain distinct and pursue their own priorities in research development. [15] Our results show some papers published after this declaration (2007 onwards) were more inclusive of Indigenous values and reported on Indigenous community involvement throughout some of the research process. However, there are still major improvements to be made in involving Indigenous voices through the whole research process. Appropriate intellectual property rights generated from the research must also reflect this and be mentioned clearly in the research outputs. [55] Furthermore, community members need to be consulted for interpreting findings and in creating a safe space for knowledge translation between Indigenous knowledge and researcher views. [54] In addition, a previous research paper on knowledge translation with Indigenous communities in Canada reported research which engages the community results in a high degree of participation and increased participation in the research process by the participants. [56] Despite the established importance of data sovereignty globally in moving Indigenous research in a positive direction, the findings from this review reveal existing research on Indigenous primary health service use has not documented research governance appropriately. Most of the papers stated appropriate ethical approvals were obtained and there was some community involvement. However, providing a more detailed description of the participant consent and ownership of data by Indigenous Peoples would demonstrate more engagement of primary health services by Indigenous Peoples. Dissemination of research outputs is an integral part of the research process to ensure the conducted research has political, social and economic impact. [57] The exchange of research findings between Indigenous stakeholders, health service, and policy makers and the dissemination plans that are inclusive of Indigenous values must be clearly outlined in the research papers. Ninomiya et al. state the social value of reporting to Indigenous stakeholders provides an effective strategy in knowledge translation and partnership. [58] This provides opportunities for Indigenous communities to utilise the information to monitor health discrepancies and advocate for policy changes and relevant resources.

A previous systematic meta-ethnographic review by De Zilva et al. (2022) included 34 studies on culturally safe healthcare practice for Indigenous Peoples in Australia. [8] Findings identified trusting relationships and supportive healthcare systems that are responsive to Indigenous People’s cultural knowledge, beliefs and values as being important for cultural safety healthcare. Another review by Poitras et al. investigated cultural safety interventions in primary care amongst urban Indigenous Peoples for chronic disease. [59] Poitras et al. revealed healthcare professionals need to be more aware of Indigenous Peoples’ history and culture and include family, appropriate visual aids, and consideration of spirituality in their practices. [59] Also, Poitras et al. emphasised the importance of involving Elders as traditional healers and guides for Indigenous Peoples to provide guidance between different spheres of holistic health, which is a facilitator for Indigenous health. [59] Whilst these interventions are based on healthcare practices, they must also be utilised in research outputs to produce research that leads to equitable access. Our findings demonstrated that whilst most papers (71.4%, 25/35) mentioned some description of applying Indigenous research methodologies, researchers need to provide more detail. Specifically in terms of providing examples of what these methodologies are and why they are important to Indigenous beliefs to ensure that research conducted is away from Western research bias. In addition to this, we found a dominance of papers reporting on PHC use for Indigenous Peoples with diabetes (more than half, 57%) and only one paper on chronic kidney disease (2.9%). This is problematic using Indigenous framework of knowing which rely on holistic models of care that consider ‘health as a whole’. Researchers need to be mindful and consider Indigenous frameworks to ensure findings are relevant to community needs and offer a holistic transfer of knowledge to community level.

### Study strengths and limitations

The key strength of this systematic review is its conduct as part of the ARDAC study. [60] As such, we have been able to ensure our research is conducted in a culturally safe way, with the ARDAC Study’s Advisory Group and Aboriginal and Torres Strait Islander investigators providing oversight of the systematic review process and guidance on the interpretation of the findings. However, the findings from this systematic review are limited by the identification of only a small number of articles from some countries. For example, only one article was eligible for inclusion from Canada. The lack of papers from some regions may reflect an absence of research on PHC use within Indigenous Communities in Canada for the specific chronic diseases in this review. Given the inclusion criteria searched specifically for Indigenous Peoples with chronic kidney disease, diabetes mellitus and/or cardiovascular disease, papers reporting on PHC use by Indigenous Peoples for chronic diseases as a whole or other chronic diseases may have been missed. Therefore, it is important to note there may be culturally safe research practices being led by Indigenous communities and/or implemented across these locations, that have not been reported in traditional academic forums and, therefore, not identified in our searches nor included in our findings. In addition, although corresponding authors for included papers were contacted, we were unable to confirm Indigenous research governance for some of the papers. As a result, our identification of Indigenous representation within the authorship lists and governance committees may be underreported. Finally, another limitation is the use of the two cultural safety assessment tools, which were developed after 2019, to assess studies which predominantly predate the development and publication of the tools. As a result, we are applying a contemporary lens of cultural safety to research conducted and published during a period when cultural safety was not present in the zeitgeist. Therefore, we acknowledge that although the included papers may not have addressed specific criteria from the CONSIDER checklist within their reporting of the research, it may have been addressed in the overall conduct of their research.

### Implications for practice, policy, and research

The findings from this study identifies several opportunities to enhance the cultural safety of Indigenous PHC, both in terms of health service practices and research outputs. This includes enhancing Indigenous research governance by providing clear statements outlining the intellectual property negotiations and partnership agreements (such as MOUs and MOAs) between Indigenous and non-Indigenous researchers. Indigenous data sovereignty needs to also be considered, and includes obtaining collective consent from research participants, especially in terms of further analysis and storage of any data or biological samples needs to be described clearly. Integrating cultural safety into primary healthcare services allows greater equity of access and leads to preventing onset of a myriad of chronic diseases. [61] PHC services such as general practice clinics should invest in maintaining strong relationships between Indigenous stakeholders and understanding client’s needs, providing employment and training opportunities for Indigenous Peoples, and adapting flexible ways to providing care. [61, 62] Including Indigenous Peoples in the provision of primary healthcare leads to improved communication between patients and carers and continuity of care. For example, a qualitative study on cancer care provision reported collaborative approaches, patient-centred care and timely communication and information exchange were crucial in improving quality cancer care for Indigenous Australians. [63] Whilst based on cancer healthcare, the findings are transferrable to chronic disease care for Indigenous peoples in that collaborative approaches and patient centred care leads to improved quality of care. [63] Governments should also follow recommendations provided by research outputs and invest in PHC services underpinned by Community values and principles.

## Conclusions

Indigenous PHC must adopt more culturally safe ways of providing care and producing research outputs which are relevant to Community needs. Given that PHC services are the frontline for healthcare delivery, privileging Indigenous voices in the conduct and reporting of research enhances the acceptability of research findings within communities. Previous literature has emphasised the importance of Indigenous Peoples’ involvement in research and health service practices related to their health. Indigenous stakeholders must be involved throughout the research process to guide the practices in a positive direction that is inclusive of Indigenous values and are informed by community needs. Governments, policy makers and other relevant stakeholders should invest in more employment and training for Indigenous Peoples to be involved in PHC settings to increase access and reduce the burden of chronic disease.

### Electronic supplementary material

Below is the link to the electronic supplementary material.

Supplementary Material 1

Supplementary Material 2

## Data Availability

All data generated or analysed during this study are included in this published article [and its supplementary information files].

## Abbreviations

PHC: Primary Healthcare
AMSTAR: Assessing the Methodological quality of Systematic Reviews
CONSIDER: The Consolidated Criteria for Strengthening Reporting of Health Research Involving Indigenous Peoples
CREATE: using the Aboriginal and Torres Strait Islander Quality Appraisal
MOU: Memorandum of Understanding
MOA: Memorandum of Agreement
