# Supplementary material for: The cultural safety of research reports on primary healthcare use by Indigenous Peoples: a systematic review
Source: BMC Health Serv Res. 2024 Jul 31;24:873. doi: 10.1186/s12913-024-11314-3 (PMC11293170; doi:10.1186/s12913-024-11314-3)
Supplement: Supplementary file 1 — Supplementary Material 1 [file 12913_2024_11314_MOESM1_ESM.docx]

**Supplementary File 1:**

**Supplementary Analysis using CREATE tool**

**Background**

The Aboriginal and Torres Strait Islander quality appraisal tool, also known as the CREATE tool was used to undertake a subset analysis of the reporting of only the Australian papers (n = 20) to strengthen the evidence-base for current reporting of PHC research on Indigenous Peoples. The decision to use this tool for only the Australian papers was made given that this tool was created specifically for Australian studies and therefore, was not appropriate to use for the other countries. The CREATE tool was developed by a South Australian research group of 11 researchers with expertise in public health, ethics, biomedical and clinical research, and systemic reviews. Of the 11 researchers, six were senior Aboriginal and Torres Strait Islander researchers and five were non-Indigenous researchers. The CREATE tool was developed to address the gap of which assess the quality of health research from an Aboriginal and Torres Strait Islander perspective. The CREATE tool consists of 14 appraisal questions developed using a modified Nominal group and Delphi Techniques to assess the rigour and appropriateness of method. Where data was missing or unclear the researchers contacted the corresponding author to retrieve additional information. The results from the supplementary analysis using the CREATE tool are displayed in supplementary figure 1 (see below).

**
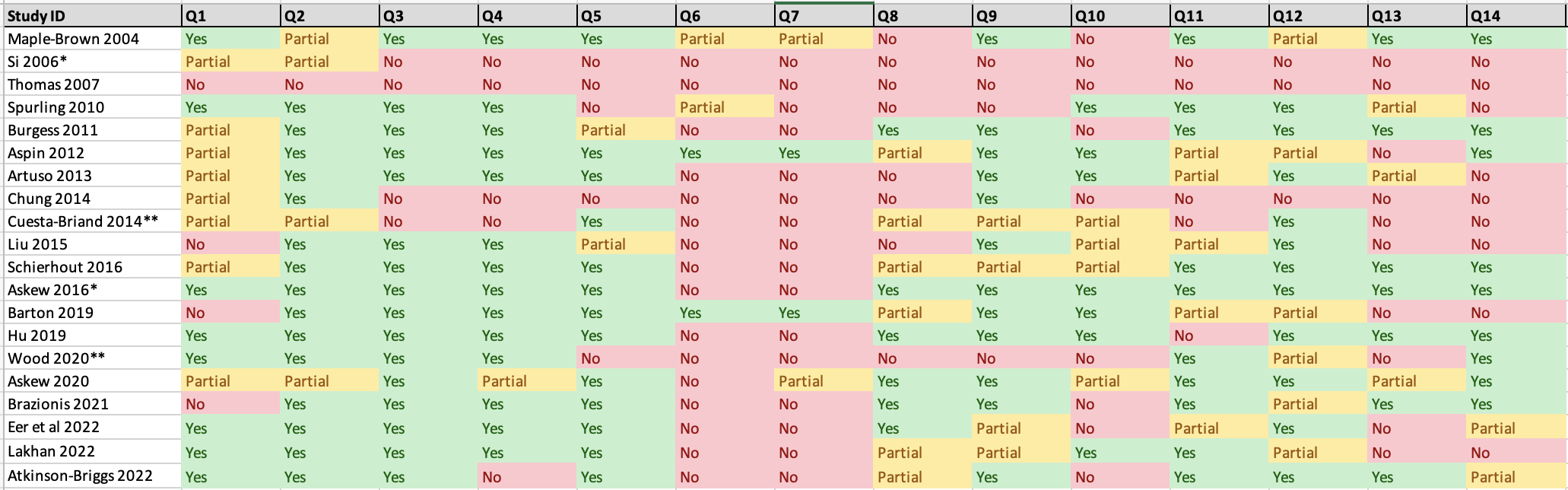
Results:**

**Supplementary Material 1 Figure 1**- Cultural safety assessment of Australian papers (n = 20) using the CREATE tool

All 20 Australian papers included in the systematic review was assessed using the CREATE tool. The cultural safety of these Australian papers varied particularly in terms of the way reporting of different aspects of cultural safety from an Aboriginal and Torres Strait Islander perspective. These aspects include items such as research governance, capacity strengthening, protection of intellectual property, Indigenous research paradigm, a strengths-based approach to research, the translation of findings into policy and practice and two-way learning. The findings are discussed below and are broken down by question.

**Question 1**

Out of the 20 papers, 40% of the papers (8/20) stated in the research output that the research was responding to a need and/or priority determined by the community. Only 15% (n = 3/20) of the papers explicitly mentioning this in the paper, by stating the research aims emerged from community needs or research aims emerged after extensive consultations with community members.

**Question 2**

Most of the papers (95%, 19/20) provided some evidence of the inclusion of community consultation and engagement throughout the research process with only one paper (5%, 1/20) lacking a detailed explanation of this in the research output. These papers stated that consultations with different communities were undertaken prior to the study.

**Question 3**

Given the recognition of Indigenous leadership in overseeing research conducted, about 80% (n = 16/20) papers reported utilising Indigenous leadership, which includes having Indigenous chief investigators or the research project being led by Aboriginal and Torres Strait Islander researchers.

**Question 4**

Most papers 75% (15/20) included reported having research governance throughout the research process. These papers stated detail about involvement of Indigenous People in informing, monitoring and guiding the direction of the project.

**Question 5**

More than half of the papers (75%, 15/20) stated that local community protocols such as the researchers familiarising themselves with appropriate community protocols, researchers being respectful of community events, and aligning data collection appropriately were respected and followed during the research.

**Question 6**

Many of the papers (90%, 18/20) did not report negotiations or agreements regarding the rights of access and protection of Indigenous Peoples’ ownership of intellectual and cultural property created through the research. Reporting of this may have included things such as providing some detail about any Memoranda of Understanding (MOUs) are commonly used to document agreements about the rights and responsibilities of the partners in research.

**Question 7**

Only 80% (16/20) of the papers provided reported Indigenous communities having control over the collection and management of research materials, where approval was sought from appropriate Indigenous organisations prior to dissemination of any findings.

**Question 8**

Most papers (60%, 12/20) demonstrated that the research was guided by an Indigenous research paradigm which refers to research that reflects on Indigenous community values, priorities, and perspectives.

**Question 9**

Most of the papers (80%, 16/20) reported being guided an Indigenous research paradigm which includes clear description of how the research reflections Aboriginal and Torres Strait Islander ways of knowing, being and doing as well as placing values on Indigenous knowledges.

**Question 10**

Just over half of the papers (55%, 11/20) took a strengths-based approach by acknowledging and moving beyond practices which have harmed Aboriginal and Torres Strait Islander peoples in the past with only 35% (7/20) clearly stating this in the research outputs. This includes mention of ongoing colonising practices, and systemic racism and moving away from deficit discourse.

**Question 11**

More than half of the papers (75%, 15/20) reported a plan to translate the findings into sustainable changes in policy and/or practice. This includes any evidence of a comprehensive knowledge-translation plan in the research outputs evidence which demonstrates the research has resulted in policy development or informed practice.

**Question 12**

Majority of the papers (90%, 18/20) reported that the research conducted benefited the participants and Aboriginal and Torres Strait Islander communities. This was particularly more evident in more recent papers (2014-15 onwards).

**Question 13**

Capacity strengthening for Aboriginal and Torres Strait Islander peoples who are members of the participating communities is essential in improving their health and wellbeing. Half of the papers (50%, 10/20) reported some evidence of employment or formal and/or informal training opportunities delivered to Aboriginal and Torres Strait Islander peoples as part of the research process.

**Question 14**

Just over half of the papers (55%, 11/20) had some mention of two-way learning in the research outputs where everyone involved in the study has opportunities to learn from each other. Overall, more recent papers (2015 onwards) have conducted research that is more inclusive of Indigenous values and has also included Indigenous Peoples throughout the research process by facilitating capacity-strengthening opportunities as well.
