# Supplementary material for: The cultural safety of research reports on primary healthcare use by Indigenous Peoples: a systematic review
Source: BMC Health Serv Res. 2024 Jul 31;24:873. doi: 10.1186/s12913-024-11314-3 (PMC11293170; doi:10.1186/s12913-024-11314-3)
Supplement: Supplementary file 2 — Supplementary Material 2 [file 12913_2024_11314_MOESM2_ESM.docx]

**Supplementary File 2:**

**Search Terms**

| **MEDLINE SEARCH** | |
| --- | --- |
|  |  |
| 1 | american native continental ancestry group/ |
| 2 | indians, central American/ |
| 3 | indians, north American/ |
| 4 | alaskan natives/ |
| 5 | indigenous canadians/ |
| 6 | american natives/ |
| 7 | oceanic ancestry group/ |
| 8 | (Aboriginal* or Torres Strait Islander or ATSI or first nation* or indigenous or first people* or Kaurna or Adnyamathanha or Mula or Maralinga or Narungga or Ngaanyatjarra or Ngarrindjeri or Pitjantjatjara or Yolngu or Anangu or Yankunytjatjara or Arrernte or Aranda or Arunta or Arrarnta or Inuit* or Inupiat* or Metis* or Metis* or Kalaallit* or aleut* or eskimo* or Whenua* or kiwi* or Maori* or Maori).tw,kf. |
| 9 | ((Austra* or New Zealand or Aotearoa* or Pacific island* or Hawaii* or American or Canad*) adj3 (race* or native*)).tw,kf. |
| 10 | 1 or 2 or 3 or 4 or 5 or 6 or 7 or 8 or 9 |
| 11 | Chronic Disease/ |
| 12 | ((chronic or endstage* or end-stage* or disease*) adj3 (renal or kidney or cardiovascular or disease* or pulmonary or heart or coronary)).tw,kf. |
| 13 | kidney diseases/ |
| 14 | diabetes insipidus/ |
| 15 | diabetic nephropathies/ |
| 16 | exp hypertension, renal/ |
| 17 | exp nephritis/ |
| 18 | (hemodialysis or haemodialysis or dialysis or hemofiltration or haemofiltration or CKD or ESKD or nephropath* or nephrit* or glomerulo* or glomerular disease* or predialysis or pre-dialysis or renal hyperten*).tw,kf. |
| 19 | exp Coronary Disease/ |
| 20 | exp Myocardial Ischemia/ |
| 21 | exp Atherosclerosis/ |
| 22 | (myocard* adj3 (infarct* or ischemi* or coronary artery disease)).tw,kf. |
| 23 | (angina or heart attack* or atherosclero*).tw,kf. |
| 24 | Diabetes Mellitus, Type 2/ |
| 25 | Diabetes Insipidus, Neurogenic/ |
| 26 | Diabetes Mellitus, Type 1/ |
| 27 | Diabetes Mellitus/ |
| 28 | (diabetes or diabetic or T2DM or T1DM).tw,kf. |
| 29 | 11 or 12 or 13 or 14 or 15 or 16 or 17 or 18 or 19 or 20 or 21 or 22 or 23 or 24 or 25 or 26 or 27 or 28 |
| 30 | Primary Health Care/ |
| 31 | Community Health Nursing/ or Family Nursing/ or Community Health Services/ or Community Health Workers/ or Nurse Practitioners/ or Family Nurse Practitioners/ or Physicians, Family/ |
| 32 | (primary health care or primary health-care or primary care or primary care or phc or PCP).tw,kf. |
| 33 | (General practic* or GP).tw,kf. |
| 34 | (715 health check or 715 health-check).tw,kf. |
| 35 | (health visit* or health-visit).tw,kf. |
| 36 | (Community adj2 (health* or nurs*)).tw,kf. |
| 37 | ((Family or (physician* or practice or doctor*))).tw,kf. |
| 38 | (((health OR patient) adj2 (worker* OR navigator* OR "liaison officer" OR practitioner OR preceptor*))).tw,kf. |
| 39 | (peer navigator* or Community reference group* or community-controlled organisations or ACCHOs).tw,kf. |
| 40 | 31 or 32 or 33 or 34 or 35 or 36 or 37 or 38 |
| 41 | 10 and 30 and 40 |
| 42 | limit 41 to yr="2002 -Current" |

| **CINAHL SEARCH** | |
| --- | --- |
| S1 | (MH "Indigenous Peoples+") |
| S2 | TI ( Aboriginal* OR "Torres Strait Islander" OR ATSI OR "first nation*" OR indigenous OR "first people*" OR Kaurna OR Adnyamathanha OR Mula OR Maralinga OR Narungga OR Ngaanyatjarra OR Ngarrindjeri OR Pitjantjatjara OR Yolngu OR Anangu OR Yankunytjatjara OR Arrernte OR Aranda OR Arunta OR Arrarnta OR Inuit* OR Inupiat* OR Metis* OR Metis* OR Kalaallit* OR aleut* OR eskimo* OR Whenua* OR kiwi* OR Maori* OR Maori ) OR AB ( Aboriginal* OR "Torres Strait Islander" OR ATSI OR "first nation*" OR indigenous OR "first people*" OR Kaurna OR Adnyamathanha OR Mula OR Maralinga OR Narungga OR Ngaanyatjarra OR Ngarrindjeri OR Pitjantjatjara OR Yolngu OR Anangu OR Yankunytjatjara OR Arrernte OR Aranda OR Arunta OR Arrarnta OR Inuit* OR Inupiat* OR Metis* OR Metis* OR Kalaallit* OR aleut* OR eskimo* OR Whenua* OR kiwi* OR Maori* OR Maori ) |
| S3 | TI ( ((Austra* OR New Zealand OR Aotearoa* OR Pacific island* OR Hawaii* OR American OR Canad*) N2 (race* OR native*)) ) OR AB ( ((Austra* OR New Zealand OR Aotearoa* OR Pacific island* OR Hawaii* OR American OR Canad*) N2 (race* OR native*)) ) |
| S4 | S1 OR S2 OR S3 |
| S5 | (MH "Chronic Disease") |
| S6 | TI ( ((chronic OR endstage* OR end-stage* OR disease*) N2 (renal OR kidney OR cardiovascular OR disease* OR pulmonary OR heart OR coronary)) ) OR AB ( ((chronic OR endstage* OR end-stage* OR disease*) N2 (renal OR kidney OR cardiovascular OR disease* OR pulmonary OR heart OR coronary)) ) |
| S7 | (MH "Diabetes Insipidus+") OR (MH "Hypertension, Renal+") OR (MH "Nephritis+") |
| S8 | (MH "Dialysis+") |
| S9 | TI ( (hemodialysis OR haemodialysis OR dialysis OR hemofiltration OR haemofiltration OR CKD OR ESKD OR nephropath* OR nephrit* OR glomerulo* OR ""glomerular disease*"" OR predialysis OR ""pre-dialysis"" OR ""renal hyperten*"") ) OR AB ( (hemodialysis OR haemodialysis OR dialysis OR hemofiltration OR haemofiltration OR CKD OR ESKD OR nephropath* OR nephrit* OR glomerulo* OR ""glomerular disease*"" OR predialysis OR ""pre-dialysis"" OR ""renal hyperten*"") ) |
| S10 | (MH "Cardiovascular Diseases+") |
| S11 | TI ( ((myocard* OR coronary OR Heart) N2 (infarct* OR ischemi* OR disease)) ) OR AB ( ((myocard* OR coronary OR Heart) N2 (infarct* OR ischemi* OR disease)) ) " |
| S12 | TI ( (angina OR 'heart attack*' OR atherosclero*) ) OR AB ( (angina OR 'heart attack*' OR atherosclero*) ) |
| S13 | (MH "Diabetes Mellitus+") |
| S14 | TI ( (diabetes OR diabetic OR T2DM OR T1DM) ) OR AB ( (diabetes OR diabetic OR T2DM OR T1DM) ) |
| S15 | S5 OR S6 OR S7 OR S8 OR S9 OR S10 OR S11 OR S12 OR S13 OR S14 |
| S16 | (MH "Primary Health Care") |
| S17 | (MH "Community Health Nursing") OR (MH "Family Nursing") OR (MH "Community Health Workers") OR (MH "Community Health Services") OR (MH "Nurse Practitioners") OR (MH "Adult Nurse Practitioners") OR (MH "Family Nurse Practitioners") OR (MH "Physicians, Family") |
| S18 | TI ( ("primary health care" OR "primary health-care" OR "primary care" OR "primary care" OR phc OR PCP) ) OR AB ( ("primary health care" OR" primary health-care" OR "primary care" OR "primary care" OR phc OR PCP) ) |
| S19 | TI ( ("General practic*" OR GP) ) OR AB ( ("General practic*" OR GP) ) |
| S20 | TI ( "715 health check" OR "715 health-check" ) OR AB ( "715 health check" OR "715 health-check" ) |
| S21 | TI ( ("health visit*" OR "health-visit") ) OR AB ( ("health visit*" OR "health-visit") ) |
| S22 | TI ( (Community N2 (health* OR nurs*)) ) OR AB ( (Community N2 (health* OR nurs*)) ) |
| S23 | TI ( (Family OR (physician* OR practice OR doctor*)) ) OR AB ( (Family OR (physician* OR practice OR doctor*)) ) |
| S24 | TI ( ((health OR patient) N2 (worker* OR navigator* OR "liaison officer" OR practitioner OR preceptor*)) ) OR AB ( ((health OR patient) N2 (worker* OR navigator* OR "liaison officer" OR practitioner OR preceptor*)) ) |
| S25 | TI ( "" peer navigator*"" OR ""Community reference group*"" OR ""community-controlled organisations"" OR ACCHOs ) OR AB ( "" peer navigator*"" OR ""Community reference group*"" OR ""community-controlled organisations"" OR ACCHOs )" |
| S26 | S17 OR S18 OR S19 OR S20 OR S21 OR S22 OR S23 OR S24 OR S25 |
|  | S4 AND S15 AND S26 |
|  | S4 AND S16 AND S27  Limiters - Published Date: 20020101-20220431 |

| **EMBASE SEARCH** | |
| --- | --- |
|  |  |
| 1 | American Indian/ |
| 2 | Alaska native/ |
| 3 | Canadian Aboriginal/ |
| 4 | oceanic ancestry group/ |
| 5 | (Aboriginal* or Torres Strait Islander or ATSI or first nation* or indigenous or first people* or Kaurna or Adnyamathanha or Mula or Maralinga or Narungga or Ngaanyatjarra or Ngarrindjeri or Pitjantjatjara or Yolngu or Anangu or Yankunytjatjara or Arrernte or Aranda or Arunta or Arrarnta or Inuit* or Inupiat* or Metis* or Metis* or Kalaallit* or aleut* or eskimo* or Whenua* or kiwi* or Maori* or Maori).tw,kf. |
| 6 | ((Austra* or New Zealand or Aotearoa* or Pacific island* or Hawaii* or American or Canad*) adj3 (race* or native*)).tw,kf. |
| 7 | 1 or 2 or 3 or 4 or 5 or 6 |
| 8 | Chronic Disease/ |
| 9 | ((chronic or endstage* or end-stage* or disease*) adj3 (renal or kidney or cardiovascular or disease* or pulmonary or heart or coronary)).tw,kf. |
| 10 | kidney diseases/ |
| 11 | diabetes insipidus/ |
| 12 | diabetic nephropathy/ |
| 13 | renovascular hypertension/ |
| 14 | exp nephritis/ |
| 15 | (hemodialysis or haemodialysis or dialysis or hemofiltration or haemofiltration or CKD or ESKD or nephropath* or nephrit* or glomerulo* or glomerular disease* or predialysis or pre-dialysis or renal hyperten*).tw,kf. |
| 16 | exp Coronary Disease/ |
| 17 | exp Myocardial Ischemia/ |
| 18 | exp Atherosclerosis/ |
| 19 | (myocard* adj3 (infarct* or ischemi* or coronary artery disease)).tw,kf. |
| 20 | (angina or heart attack* or atherosclero*).tw,kf. |
| 21 | Diabetes Mellitus, Type 2/ |
| 22 | Diabetes Insipidus, Neurogenic/ |
| 23 | Diabetes Mellitus, Type 1/ |
| 24 | Diabetes Mellitus/ |
| 25 | (diabetes or diabetic or T2DM or T1DM).tw,kf. |
| 26 | 8 or 9 or 10 or 11 or 12 or 13 or 14 or 15 or 16 or 17 or 18 or 19 or 20 or 21 or 22 or 23 or 24 or 25 |
| 27 | Primary Health Care/ |
| 28 | Community Health Nursing/ or Family Nursing/ or Community Health Services/ or Community Health Workers/ or Nurse Practitioners/ or Family Nurse Practitioners/ or Physicians, Family/ |
| 29 | (primary health care or primary health-care or primary care or primary care or phc or PCP).tw,kf. |
| 30 | (General practic* or GP).tw,kf. |
| 31 | (715 health check or 715 health-check).tw,kf. |
| 32 | (health visit* or health-visit).tw,kf. |
| 33 | (Community adj2 (health* or nurs*)).tw,kf. |
| 34 | ((Family or (physician* or practice or doctor*))).tw,kf. |
| 35 | (((health OR patient) adj2 (worker* OR navigator* OR "liaison officer" OR practitioner OR preceptor*))).tw,kf. |
| 36 | (peer navigator* or Community reference group* or community-controlled organisations or ACCHOs).tw,kf. |
| 37 | 27 or 28 or 29 or 30 or 31 or 32 or 33 or 34 or 35 or 36 |
| 38 | 7 and 26 and 37 |
| 39 | limit 37 to yr="2002 -Current" |
